# Radiolysis *via* radioactivity is not responsible for rapid methane oxidation in subterranean air

**DOI:** 10.1371/journal.pone.0206506

**Published:** 2018-11-01

**Authors:** Arndt Schimmelmann, Angel Fernandez-Cortes, Soledad Cuezva, Thomas Streil, Jay T. Lennon

**Affiliations:** 1 Department of Earth and Atmospheric Sciences, Indiana University, Bloomington, Indiana, United States of America; 2 Department of Biology and Geology, University of Almeria, Almeria, Spain; 3 Department of Earth Sciences, Royal Holloway, University of London, Egham, Surrey, United Kingdom; 4 SARAD GmbH, Dresden, Germany; 5 Department of Biology, Indiana University, Bloomington, Indiana, United States of America; University of California Santa Barbara, UNITED STATES

## Abstract

Atmospheric methane is rapidly lost when it enters humid subterranean critical and vadose zones (e.g., air in soils and caves). Because methane is a source of carbon and energy, it can be consumed by methanotrophic methane-oxidizing bacteria. As an additional subterranean sink, it has been hypothesized that methane is oxidized by natural radioactivity-induced radiolysis that produces energetic ions and radicals, which then trigger abiotic oxidation and consumption of methane within a few hours. Using controlled laboratory experiments, we tested whether radiolysis could rapidly oxidize methane in sealed air with different relative humidities while being exposed to elevated levels of radiation (more than 535 kBq m^-3^) from radon isotopes ^222^Rn and ^220^Rn (i.e., thoron). We found no evidence that radiolysis contributed to methane oxidation. In contrast, we observed the rapid loss of methane when moist soil was added to the same apparatus in the absence of elevated radon abundance. Together, our findings are consistent with the view that methane oxidizing bacteria are responsible for the widespread observations of methane depletion in subterranean environments. Further studies are needed on the ability of microbes to consume trace amounts of methane in poorly ventilated caves, even though the trophic and energetic benefits become marginal at very low partial pressures of methane.

## Introduction

Energetic radiation generates ions and radicals in fluids *via* radiolysis that can trigger subsequent chemical reactions [1], including the oxidation of organics. Radiolysis has likely affected the evolution of early microbial metabolisms and is crucial for powering the deep microbial biosphere [2, 3]. However, few studies have addressed the quantitative importance of radiolysis for contemporary fluxes in the atmosphere and the critical zone, especially in comparison to processes that compete with biologically mediated transformations.

The concentration of methane (CH_4_) in the atmosphere has more than doubled since 1850 to ~1.85 ppmv (i.e., parts per million by volume) and now contributes ~15% of anthropogenic forcing of climate change [4]. The Intergovernmental Panel on Climate Change (IPCC) report [5] includes secondary greenhouse warming effects of CH_4_ and arrives at 1 W m^-2^ for CH_4_ relative to 1.7 W m^-2^ for CO_2_, making CH_4_ the second most important anthropogenic climate forcing agent. In the atmosphere, the removal of CH_4_ is due primarily to oxidation *via* photochemically generated tropospheric OH• radicals ([6], and refs. therein). In spite of intense radiation in the atmosphere from sun and space, the residence time of atmospheric CH_4_ is ~12 years. The second largest sink for atmospheric CH_4_ is shallow subterranean environments containing aerated soils that are inhabited by CH_4_ oxidizing bacteria, MOB [4, 7]. MOB are also found in deeper aerated subterranean environments, such as caves in the vadose zone, although their contribution to global CH_4_ cycling has not been quantified or incorporated into earth system models [4].

A growing number of studies have reported that, throughout the world, concentrations of CH_4_ are often depleted in the air of caves suggesting that subterranean environments may represent an overlooked sink for atmospheric CH_4_ (e.g., [8–13]). Based on ventilation rates and CH_4_ pools, it is estimated CH_4_ is rapidly consumed in caves on time scales ranging from hours to days [14, 15]. Depletion of CH_4_ in caves is often attributed to MOB. However, a study from Spanish caves proposed that rapid CH_4_ oxidation may be attributed to non-biological processes *via* radiolysis and ionization of subterranean air by natural radioactivity that could lead to the oxidation of CH_4_ at a sufficiently fast rate to account for appreciable consumption of CH_4_ [10]. It has been proposed that α-radiation (e.g., from ^222^Rn) can radiolytically ionize, or generate radicals from, atmospheric components (e.g., H_2_O) including CH_4_ [16, 17]. The study by Haynes and Kebarle [16] determined that α-radiation has a slow effect on pure CH_4_ and mixed hydrocarbon gas in the absence of air, making it difficult to extrapolate results to CH_4_ in air in the presence of ions and radicals from heteromolecules.

Some studies, however, have raised questions about the relative importance of abiotic CH_4_ oxidation based on theoretical considerations of kinetics, the inability of α-radiation from metallic uranium and radon to trigger fast oxidation of CH_4_ [15, 18]. Laboratory and field experiments implicated MOB with the rapid decline in cave CH_4_ concentrations [18], while isotopically uncharacterized radon was unable to remove CH_4_ from air in an Australian cave [15]. Studies on radon typically focus on ^222^Rn because its longer half-life of 3.83 days facilitates quantification. No study has yet examined the radiolytic effect on CH_4_ oxidation of the relatively more energetic decay of ^220^Rn (called thoron, with a half-life 55.6 s), particularly in the air close to cave walls and floors where ^220^Rn is relatively more abundant. Also, direct experiments linking the constraints of air humidity and natural radiation from specific radon isotopes to CH_4_ oxidation in air are lacking. The current study fills these gaps with detailed independent experiments in two laboratories using energetically distinct radiation levels from isotopes of radon (^222^Rn and ^220^Rn) at different humidities and contrasting the results with CH_4_-depletion by MOB.

## Materials and methods

The authors of this study belong to two teams that had no knowledge of each others’ experiments at Indiana University (IU) and Royal Holloway University of London (RHUL). After completion of all experiments, the two groups decided to jointly report their complementary results. Work at IU afforded superior analytical control on radon isotopes and could accurately measure higher dose rates, whereas the more gas-tight experimental setup at RHUL provided more straightforward evidence for the inability of natural radiation levels to rapidly oxidize atmospheric CH_4_ at its natural atmospheric abundance.

Details of materials and methods are available from *protocols*.*io* under http://dx.doi.org/10.17504/protocols.io.s7aehie. We employed two separate, complementary experimental approaches at IU and RHUL. The following two sections offer brief overviews.

### Apparatus at IU for active, time-resolved measurements of gas concentrations with circular flow

**Fig 1:**
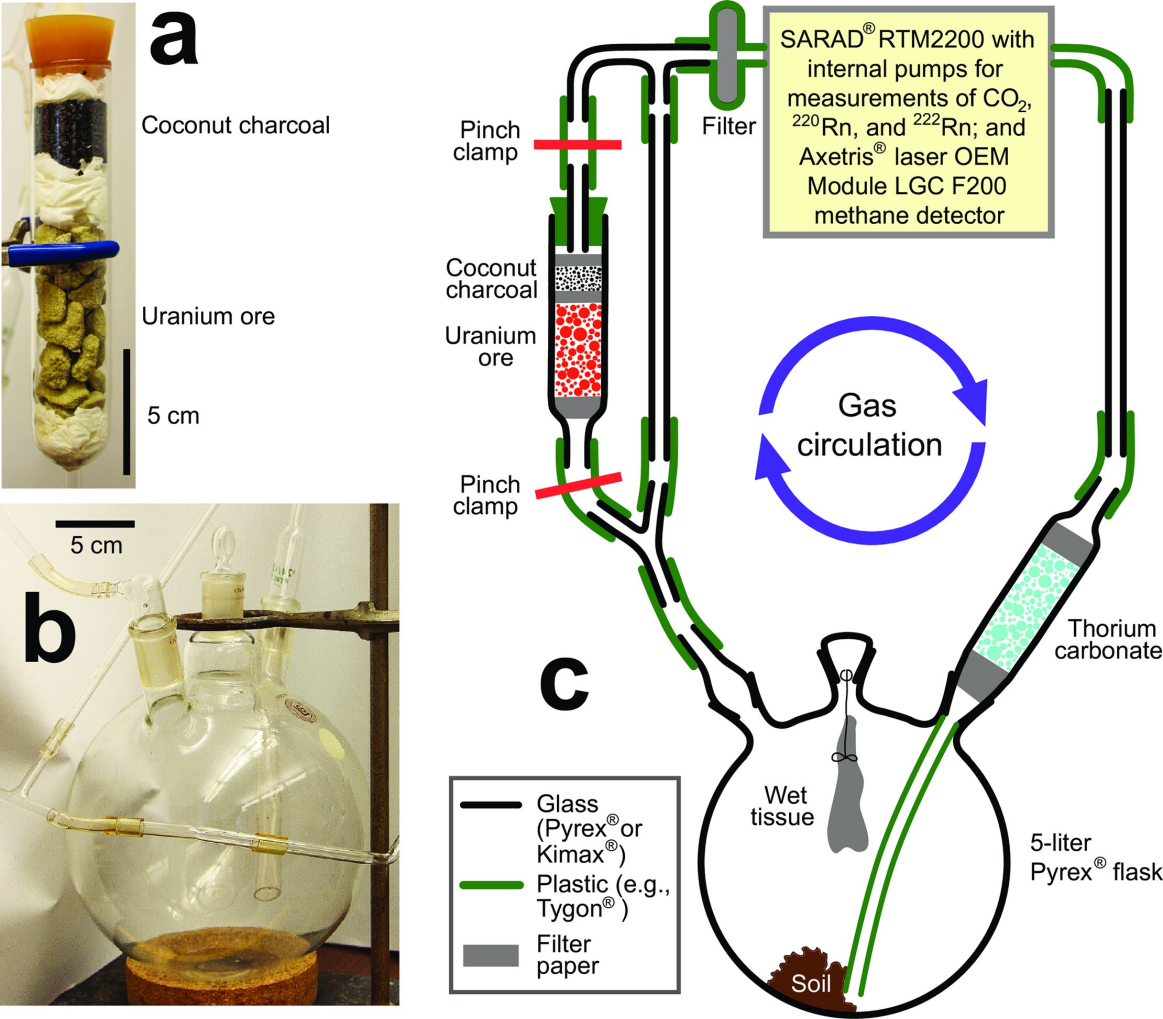
Experimental setup for time-resolved measurements of gas concentrations with circular flow at Indiana University. Approximately 6 L of air was recirculated in a sealed apparatus to assess the loss of CH_4_ with or without added radiation from radon isotopes and their radioactive progeny. At the beginning of each experiment, the trapped air was slightly enriched in CH_4_ (and CO_2_, except for experiments with soils), followed by hourly measurements of gas concentrations over a few days to weeks. Radon ^222^Rn was generated by uranium ore while charcoal retained ^220^Rn (**a**). The air intake of the 3-neck 5-L glass flask was directed to the bottom of the flask with a plastic insert to facilitate the mixing of air (**b**); thorium carbonate is not shown. Depicted components of the apparatus (**c**) are not drawn to scale.

At IU, we constructed an experimental apparatus to assess the loss of CH_4_ in an active (i.e., with pumping of air) and time-resolved manner with or without added radiation from radon isotopes and their progeny (Fig 1). The use of pumping qualifies this method as active and time-resolved in contrast to passive measurements of radon that integrate over time [19]. Approximately 6 L of air was recirculated in the sealed apparatus that included (i) a glass tube with optional thorium carbonate to generate ^220^Rn (also called thoron), and (ii) a glass tube containing uranium ore to generate ^222^Rn, with an overlying layer of coconut charcoal to limit the escape of co-produced, short-lived ^220^Rn. Blank experiments without elevated radiation identified a reproducible loss of CH_4_ (likely by diffusion through polymer tubing within the sealed analytical SARAD RTM 2200 instrument) that was subtracted from all other experiments at IU to arrive at net CH_4_ losses that are due to other factors, such as radiolysis or microbial methanotrophy.

We conducted a number of experiments at IU to assess the importance of α-radiation intensity, relative humidity, and the presence or absence of soil on CH_4_ dynamics. Moisture is critical for the emanation efficiency of radon isotopes from solid sources (i.e., the escape of noble gas radon atoms from the interior of minerals into H_2_O-containing pore space *via* recoil subsequent to radioactive decay of parental nuclides; e.g., [20, 21]) and for stabilizing ions and radicals in air. Individual experiments differed in terms of their optional use of elevated humidity, thorium carbonate, and gas flowing through the tube containing uranium ore. The trapped ~6-L volume of air was initially spiked with CH_4_ from natural gas to ~70 ppmv and with CO_2_ to ~5,000 ppmv (except for experiments with soils) to distinguish it from room air and to increase the analytical precision during the time-series of measurements that lasted over a few days to weeks. Elevated CO_2_ concentrations are typical for many cave environments [10].

Most experiments at IU discriminated between α-radiation from radon ^222^Rn *versus* thoron ^220^Rn. Whereas radon ^222^Rn with a half-life of 3.83 days is relatively homogeneously distributed in cave air (also in our apparatus), the much shorter lived thoron ^220^Rn with a half-life of only 55.6 s [22] cannot travel far from its parent nuclei residing in minerals [19], thus thoron’s highest concentrations in cave air are near cave walls and the floor. The higher α-decay energy of ^220^Rn (6.3 MeV) relative to ^222^Rn (5.49 MeV) prompted us to design experiments for separate examinations of the ability of both radon isotopes to trigger the oxidation of CH_4_. The more energetic α-decay of thoron ^220^Rn should ionize air more efficiently than ^222^Rn. Thoron was generated from thorium carbonate that was optionally loaded into a glass tube attached to the round-bottom flask. In other experiments, ^222^Rn decay measuring up to 327 kBq m^-3^ was produced *in-situ* in the glass apparatus by uranium ore chips (Fig 1). Escape of co-produced thoron from ore was reduced by using a layer of coconut charcoal in the upper part of the glass tube as a filter [23]. The resulting adsorption of ^220^Rn on charcoal increased the residence time in the glass tube and let ^220^Rn decay before it could enter the 5-L glass flask.

We quantified the concentrations of ^222^Rn, ^220^Rn, CH_4_ and CO_2_ during experiments at IU at an air flow rate of ~0.2 L min^-1^ once every hour while operating the diffusion pump in the SARAD RTM 2200. ^220^Rn radiation intensity was either measured *via* α-spectroscopy at a faster flow rate of 1 L min^-1^ in 10-min increments (n ≥ 10) while temporarily operating the more powerful membrane pump, or values from flow rates ≤0.2 L min^-1^ with the diffusion pump were doubled to adjust for fast ^220^Rn decay (see S1 File for detailed control experiments and graphed data). Elevated relative humidity fosters the stabilization of ions in air *via* attachment to clusters of water molecules and may enhance the ability of ions to trigger oxidative degradation of CH_4_ (discussed in [10]). Therefore, at IU we recorded humidity in the apparatus along with temperature, air pressure, flow, and battery voltage on an hourly basis. The accuracy of data from the SARAD RTM 2200 was independently evaluated *via* direct comparison with a newly manufactured and factory-calibrated Thoron Scout instrument (SARAD GmbH, Dresden, Germany; details available in S1 File).

We conducted a number of experiments at IU to test for the effects of radiation and microbial activity on CH_4_ dynamics in our experimental apparatus. Multi-day time-series of data were collected in closed-circuit air reflux mode (i) as duplicated blank experiments without added radon or thoron, (ii) with enhanced ^220^Rn concentration in dry or moist air, (iii) with enhanced ^222^Rn concentration in dry or moist air, and (iv) with jointly enhanced ^220^Rn and ^222^Rn concentrations in moist air to depict an extreme scenario where cave air had a highly elevated α-radiation level. Furthermore, (v) we tested for CH_4_ oxidation after placing moist soils, which we assumed contained methanotrophic bacteria (MOB), into the 5-L glass flask, without elevated radioactivity. Certain impurities in industrially conditioned natural gas may act as MOB inhibitors, for example acetylene and carbon monoxide (p. 335 in [24]). As a precaution, the CH_4_ spikes in experiments employing two different soils were derived from gas that was collected from a natural seepage of shale gas in New York State [25]. Natural shale gas is not known to contain acetylene or carbon monoxide.

### Gas-tight terrarium experiments at RHUL

**Fig 2:**
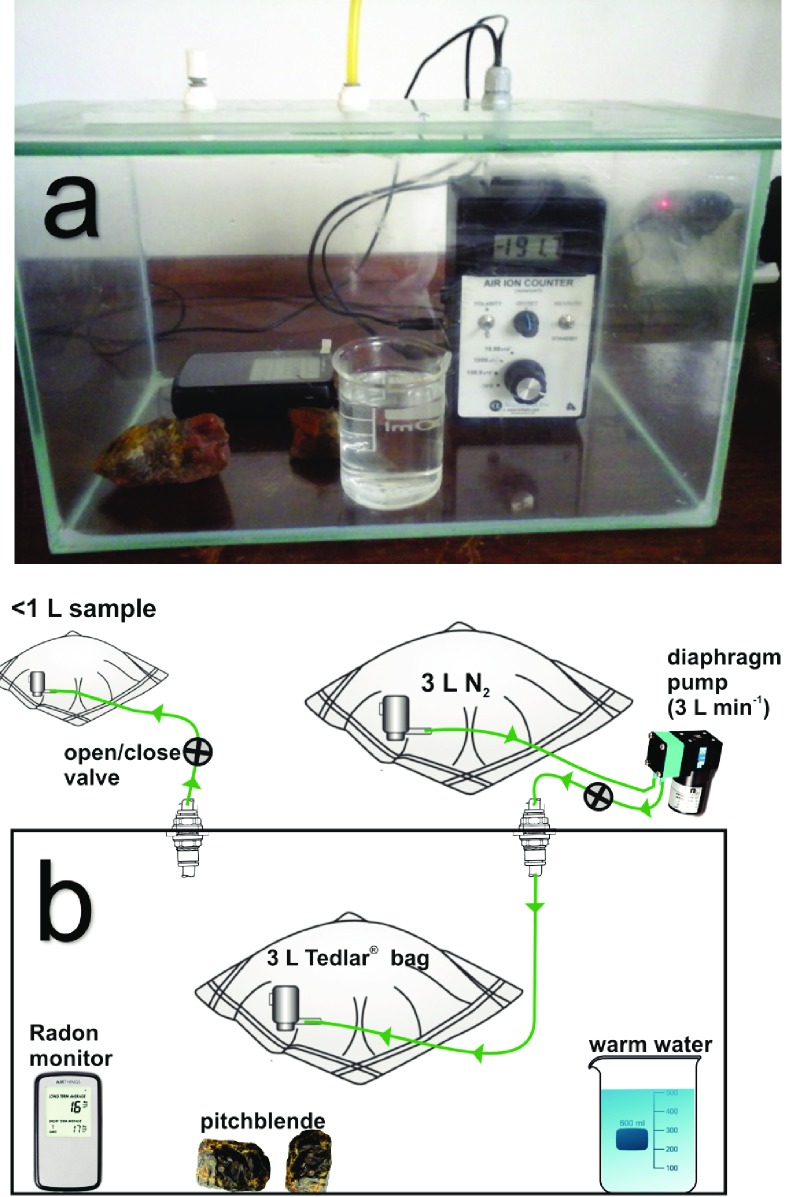
Gas-tight terrarium experiments at Royal Holloway University of London. (**a**) A hermetically sealed glass terrarium was filled with laboratory air containing atmospheric CH_4_. A radon monitor provided data on ^222^Rn abundance, while an AlphaLab Air Ion Counter measured the concentration of negative ions. After ~6 h into the RHUL experiment #1, the placement of a beaker filled with deionized, warm water elevated the relative humidity to ˃ 85%. At the same time, two fragments of pitchblende (containing uraninite as a radiation source) were placed into the terrarium to generate ^222^Rn. Tedlar bags in the terrarium are not shown in the photograph. (**b**) Diagram of the sampling procedure to collect <1-L aliquots of air from the terrarium in RHUL experiment #2. This experiment lasted for 76 h and 50 min and reached a ^222^Rn-based radiation level in excess of 50 kBq m^-3^ after 5 h.

Experiments at RHUL at atmospheric CH_4_ abundance used a gas-tight glass terrarium (i.e., an aquarium without water holding a volume of 13.45 L; Fig 2) with a hermetically sealing glass lid. Two air-tight gas ports allowed the withdrawal of 1-L air samples into Tedlar bags without changes in atmospheric pressure. Fragments of uraninite-bearing pitchblende served as a source of radioactivity. An AlphaLab Air Ion Counter with an integrated fan was placed in the terrarium to measure the abundance of ions in air in 30-s intervals. The α-radiation was quantified on 1-h intervals with a Canary Pro monitor (Airthings, Oslo, Norway) *via* α-spectrometry. Gas samples in Tedlar bags were analyzed for CH_4_ mole fractions with a Picarro G1301 CRDS (Cavity Ring-Down Spectrometer, Picarro Inc., Santa Clara, California, USA).

The initial RHUL experiment #1 (Fig 2A) assessed the production of negative ions and the abundance of ^222^Rn over ~6 h (i.e., stage 1) without either pitchblende or a beaker with water in the terrarium that had been flushed initially with laboratory air, and subsequently for ~15 h in the presence of pitchblende and a beaker with 130 mL of 38°C warm water in the terrarium (stage 2).

The subsequent RHUL experiment #2 (Fig 2B) in the same terrarium included monitoring of the CH_4_ mole fraction of laboratory air sealed in the terrarium where pitchblende and a beaker with 130 mL of water (initially at 38°C) had been placed to provide for elevated radioactivity and relative humidity. Elevated relative humidity was needed to simulate cave conditions. The AlphaLab Air Ion Counter failed to provide useful data due to static interference with Tedlar bags. The second experiment lasted for 76 h and 50 min and reached a ^222^Rn-based radiation level in excess of 50 kBq m^-3^ after 5 h. Approximate 1-L aliquots of air sampled from the terrarium were analytically compared with aliquots of exterior laboratory air on four occasions.

## Results and discussion

### Active time-series measurements with circular flow at IU

**Fig 3:**
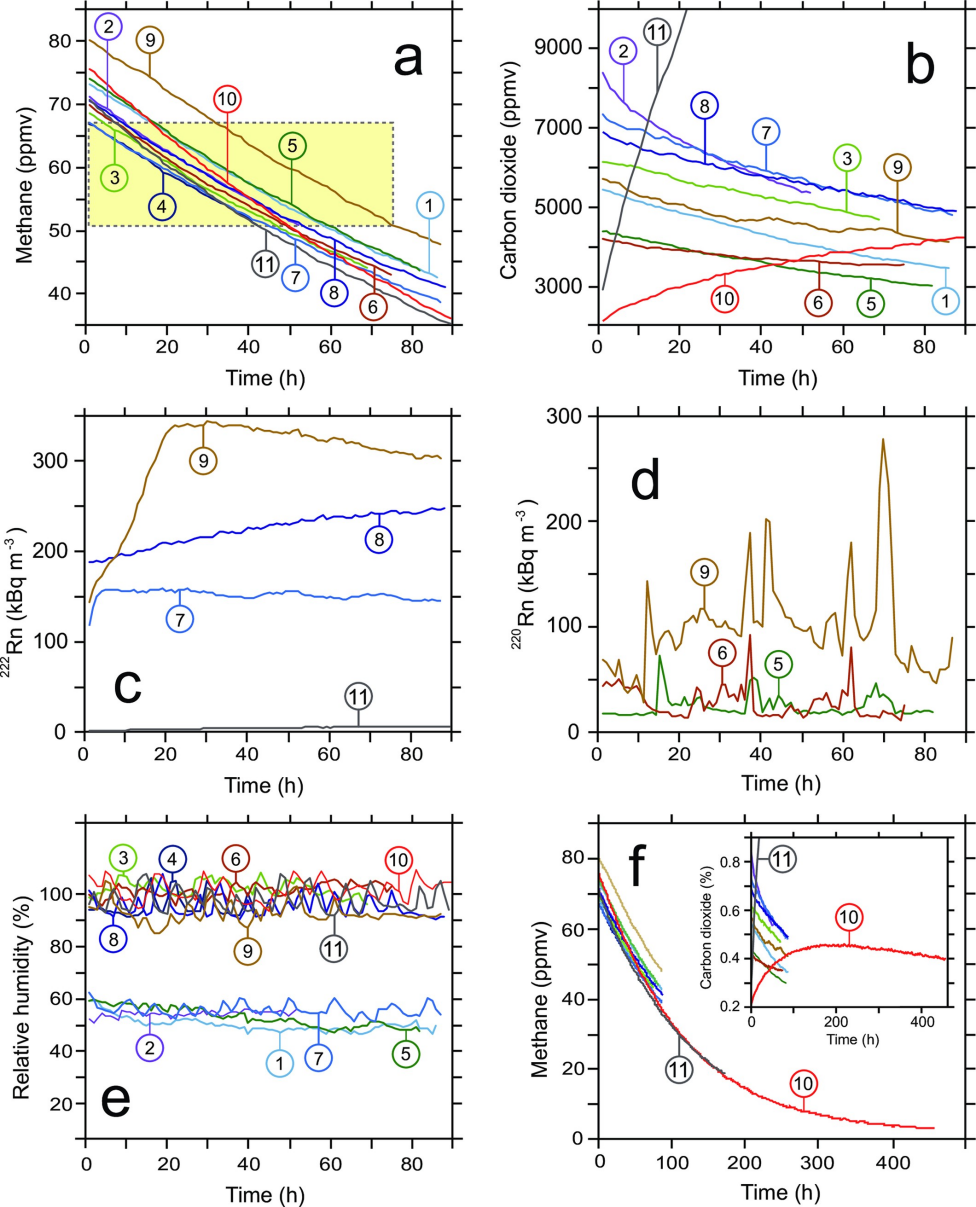
Time-series experiments at Indiana University. Results from time-series experiments to test for abiotic (radiation) and biotic (CH_4_ oxidizing bacteria) factors on CH_4_ dynamics. (**a**) The decline of CH_4_ concentrations in experiments #1 to #9 (without soil) followed similar trajectories depending on original concentrations, despite major differences in radiation intensity (see the yellow rectangle that identifies a window of CH_4_ concentrations that is common to all experiments). Declining CH_4_ concentrations are independent of the intensity of α-radiation. Blank experiments #1 to #4 without elevated radiation identify a reproducible loss of CH_4_ by diffusion that was subtracted from all other experiments to arrive at net losses that are due to other factors, such as radiolysis or microbial methanotrophy. (**b**) The decline of CO_2_ concentrations in a range of experiments without soil followed similar patterns. In addition to loss due to diffusion through plastic, it was likely influenced by adsorption, solution in water, or possible chemical uptake. In addition, CO_2_ was generated from moist soils in experiments #10 and #11. (**c**) Radon ^222^Rn and (**d**) thoron ^220^Rn concentrations partially depended on relative humidity; soil no. 2 in experiment #11 generated low levels of ^222^Rn over time presumably due to traces of uranium in minerals; ^220^Rn concentrations are original data from low flow rates at <0.2 L min^-1^ when laminar flow conditions in the 5-L glass flask caused heterogeneity and occasional spikes. (**e**) Noise in relative humidity data partially derived from the automatic battery recharge cycle that influenced the internal temperature of the SARAD RTM 2200 and the algorithm to calculate humidity. (**f**) Experiments #10 and #11 with moist soils without added ^222^Rn or ^220^Rn resulted in a long-term exponential decline of CH_4_ concentrations while CO_2_ was generated biologically.

Our controlled experiments with and without ^220^Rn and/or ^222^Rn were designed to directly test whether or not radiation can oxidize CH_4_ in cave air on ecologically relevant time scales (i.e., hours to days). We relied on comparisons of CH_4_ inventories in experiments with (i) high radiation intensity from *in-situ* generated ^220^Rn and/or ^222^Rn with those from (ii) duplicate blank experiments with no artificially enhanced radiation to demonstrate the sensitivity of our setup to detect CH_4_-losses. In addition, we conducted (iii) two experiments with moist soils in the absence of added radon isotopes to assess the potential for environmental microorganisms (i.e., MOB) to remove CH_4_ as has been demonstrated elsewhere by members of our research team [14, 18]. The comparisons among experiments covered a common range of CH_4_ concentration and thus only differed in the lengths of their time windows needed to lower the CH_4_ concentration from the upper to the lower threshold (i.e. yellow rectangle in Fig 3A). The ‘common window’ of CH_4_ decline for all 11 experiments maximized the data available for comparison.

Multiple trials in our experimental apparatus revealed that CH_4_ dynamics were unaffected by radiation within the precision of measurements. Repeat blank experiments with dry (experiments #1 and #2) or moist air (experiments #3 and #4) without artificially elevated radon or thoron concentrations resulted in reproducible and systematic small losses of both CH_4_ and CO_2_ over time (Fig 3A and 3B; Table 1A). Although radon isotopes, CH_4_ and CO_2_ could not diffuse through glass and metal in our apparatus, the SARAD RTM 2200 and its Axetris laser OEM Module LGC F200 methane detector were internally and externally connected to glass and metal components with short segments of various types of clear polymer tubing (Fig 1) that resulted in slow losses *via* gas diffusion through polymers. The rate of diffusion across a layer of polymer is dependent on the difference in partial pressures between the interior and exterior air, and hence the rates of CH_4_ and CO_2_ losses *via* diffusion over time follow curves that asymptotically approach equilibria (Fig 3F). At a CH_4_ concentration of ~59 ppmv in the apparatus (i.e., the midpoint of the common CH_4_ range; Fig 3A) and outside air with ~1.85 ppmv, the mean CH_4_ diffusive loss from air in the apparatus during blank experiments #1 to #4 consistently amounted to 0.39 ppmv h^-1^ regardless of humidity and small variations in room temperature and air pressure (Table 1A; S1 File). Such a loss of gas over time could theoretically result from a small internal leak in the system. However, the non-parallel pattern of CO_2_ losses in blank experiments (Fig 3B) is inconsistent with a leak and instead argues for varying diffusivity of the polar molecule CO_2_ through permeable material at different humidities. The observed degree of CH_4_ loss from the system was unavoidable and had to be subtracted from the observed bulk CH_4_ losses in experiments with enhanced radiation and soils to arrive at any specific losses that are due to radioactivity or presumed microbial methanotrophy.

**Table 1:** Overview on individual experiments performed at Indiana University to constrain the consumption of methane over time.

Experiment #	Overall duration (h)	Common window (h)	Mean values across common window of CH_4_ decrease from 67.2 to 50.9 ppmv						
			^220^Rn (kBq m^-3^)	^222^Rn (kBq m^-3^)	CH_4_ loss (ppmv h^-1^)	CO_2_ loss (ppmv h^-1^)	Corrected temperature (°C)	Relative humidity (%)	Pressure (kPa)
***(A) Duplicated blank experiments without added radon or thoron at low or high relative humidity***									
**#1**, no added radiation, dry	86	42.0	<0.01*	<0.01	0.39 ± 0.06	25.8 ± 14.1	23.2 ± 0.9	49.7 ± 1.5	97.2 ± 0.4
**#2**, no added radiation, dry	52	42.7	<0.01*	<0.01	0.39 ± 0.07	47.6 ± 21.3	22.8 ± 0.4	54.7 ± 0.8	98.0 ± 0.1
**#3**, no added radiation, wet	69	42.5	<0.2*	~0.4	0.39 ± 0.08	22.9 ± 23.0	23.4 ± 0.7	103 ± 2.7**	98.1 ± 0.2
**#4**, no added radiation, wet	43	42.3	<0.3*	<0.2	0.39 ± 0.07	n.d.	25.4 ± 0.7	95.3 ± 4.1	99.1 ± 0.1
***(B) Experiments with enhanced*** ^***220***^***Rn and/or*** ^***222***^***Rn concentrations at low or high relative humidity***									
**#5**, ^220^Rn added, dry	82	42.4	~50*	<0.01	0.38 ± 0.07	18.4 ± 14.2	22.9 ± 0.6	53.3 ± 2.7	98.6 ± 0.2
**#6**, ^220^Rn added, wet	75	41.8	143	<0.01	0.39 ± 0.07	10.6 ± 15.0	22.8 ± 0.7	100.4 ± 2.1	98.9 ± 0.1
**#7**, ^222^Rn added, dry	87	43.8	<0.01*	153	0.37 ± 0.07	34.7 ± 35.3	24.3 ± 0.8	56.3 ± 2.2	98.8 ± 0.2
**#8**, ^222^Rn added, wet	88	44.5	<0.04*	216	0.37 ± 0.09	22.0 ± 29.9	25.1 ± 0.9	95.5 ± 4.1	98.4 ± 0.3
**#9**, ^222^Rn and ^220^Rn added, wet	87	43.5	208	327	0.37 ± 0.11	10.3 ± 27.9	25.8 ± 1.1	92.8 ± 3.4	98.4 ± 0.2
***(C) Experiments with moist soils without added radon or thoron***									
**#10**, no added radiation, 45 g soil #1,	456	33.8	<0.03*	<0.2	0.49 ± 0.09	CO_2_ was generated	24.3 ± 0.8	100.6 ± 3.9	98.6 ± 0.2
**#11**, no added radiation, 112 g soil #2	172	34.7	~0.1*	~4	0.47 ± 0.11	CO_2_ was generated	24.7 ± 0.9	97.9 ± 4.3	98.8 ± 0.1

Analytical data are mean values with standard deviations for the time window when CH_4_ concentrations declined from 67.2 to 50.9 ppmv in each experiment. This window represents the maximum range of methane concentrations that is common to all experiments. The length of time needed to deplete methane from the upper to the lower threshold across the common window (i.e., yellow rectangle in Fig 3A) was interpolated from hourly spaced data.

* ^220^Rn radiation values were doubled when measured at flow rates ≤0.2 L min^-1^ to adjust for fast ^220^Rn decay, instead of measurements without doubling of values in 10-min intervals (n ≥ 10) at a flow rate of 1 L min^-1^. See justification in S1 File.

** High values of relative humidity are affected by analytical errors in excess of standard deviation.

n.d. = not determined.

There was a comparable loss of CH_4_ in recirculating air for all experiments without soil, regardless of the absence or presence of radiation from ^220^Rn, ^222^Rn, or both ^220^Rn and ^222^Rn, in dry or moist air (Fig 3A). The time needed to cross the ‘common window’ of CH_4_ decline from 67.2 to 50.9 ppmv was not shorter when radiation from ^220^Rn and/or ^222^Rn was added (Table 1A, 1B). The slopes of lines representing CH_4_ decline within the common window in Fig 3A were not higher for experiments with elevated radiation (mean ~0.38 ppmv h^-1^) than for blank experiments without added radon isotopes (mean ~0.39 ppmv h^-1^; Table 1A and 1B). The mean levels of added radiation from ^220^Rn, and especially the cumulative radiation in experiment #9 from simultaneously added ^220^Rn and ^222^Rn, ranged between ~50 and 535 kBq m^-3^ after doubling of experimental ^220^Rn values that were measured at ≤0.2 L min^-1^ (Table 1B) and thus always exceeded the radiation levels reported in cave air [26], including the air in all Spanish caves where abiotically driven CH_4_ oxidation due to radiolysis has been reported [10]. For example, the average rate of CH_4_ consumption in Spanish Altamira Cave air of -0.03 ppmv h^-1^ occurred at a maximum ^222^Rn radiation level of ~6 kBq m^-3^, which is roughly one to three orders of magnitude less than the radiation in any of our experiments with added radon isotopes (#5 through #9). Thus, in terms of radiation intensity, our experiments represent an extreme test of the radiolysis hypothesis. Only the air in shafts of underground uranium mines has been observed to reach even higher radiation levels of one million or more Bq m^-3^ [27].

The consistent pattern of CH_4_ decline in our experiments without soils can be better appreciated in light of the observed CO_2_ dynamics (Fig 3B). CO_2_ is more polar than CH_4_, can be more easily adsorbed on surfaces, and is more water-soluble and reactive than CH_4_. Therefore, it is possible that changes in room temperature (21.1 to 27.5°C) and atmospheric pressure (96.7 to 99.3 kPa) may have affected adsorption and solubility of CO_2_ during our experiments. Moreover, after one week of measurements with a wet paper tissue in the 4-L glass flask without soil, fungi had discolored the paper tissue and metabolically generated CO_2_, thus partially stabilizing the CO_2_ partial pressure (experiment #6, Fig 3B), apparently without affecting the CH_4_ decline (Fig 3A). The paper tissue had been hung by a thread from the central glass stopcock to maximize surface area and to avoid any anoxic microenvironments that could facilitate biological methanogenesis (Fig 1). Subsequent experiments in moist air without soil replaced the wet paper tissue with added deionized water at the bottom of the 4-L glass flask. Experiments with soils initially generated CO_2_ *via* microbial and fungal remineralization of soil organic matter, followed after several days by a decline due to diffusive loss of CO_2_.

In the two experiments with moist soils, we documented a CH_4_ loss of of ~0.09 ppmv h^-1^ within the common window of CH_4_ concentration decline (Fig 3A), as determined by subtracting the diffusive CH_4_ loss in blank experiments from the bulk CH_4_ loss in experiments #10 and #11 with soils (Table 1). It is well established that heterogeneously distributed methanotrophic biofilms in the subsurface [28] are capable of scavenging CH_4_ from the atmosphere (e.g., [29, 30]). Soil gas can often reach ^222^Rn radiation levels of many thousand Bq m^-3^, depending on local geology [31, 32]. If radiolysis would indeed be able to trigger fast oxidative decay of CH_4_ in soil gas, such an important CH_4_ sink in dry soils without abundant methanotrophic activity would likely have been documented. Also, radiolysis would compete with methanotrophs in moist soils for CH_4_ and would have been identified as a factor in soil CH_4_ studies.

### Experiments in gas-tight terrarium at RHUL

**Fig 4:**
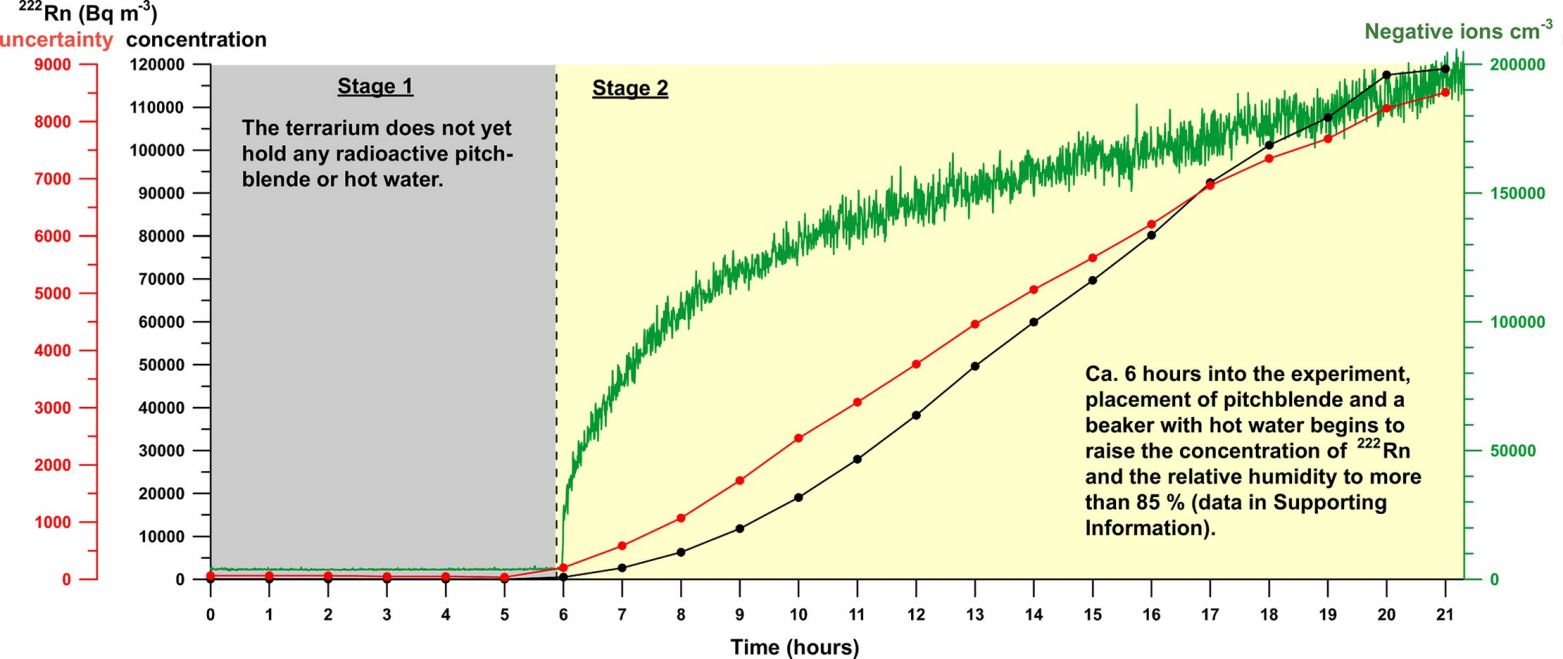
Experiment #1 at Royal Holloway University of London. The experiment proceeded in two stages where the first stage established background conditions in a hermetically closed terrarium at low relative humidity and in the absence of artificially elevated concentrations of ^222^Rn. In the second stage, the addition of pitchblende and a beaker with hot water provided a source of ^222^Rn and high humidity to simulate conditions characteristic of cave environments. Although the final ^222^Rn concentration exceeded 100 kBq m^-3^ and was thus higher than in most caves, the elevated radiation and ionization of air in the terrarium was unable to lower the atmospheric abundance of CH_4_ over 77 h in the subsequent RHUL experiment #2 (Table 2).

The first stage of experiment #1 at RHUL (Figs 2A and 4) established background conditions for the abundance of negative ions (~3800 ions cm^-3^) and the concentration of ^222^Rn (17 to 51 Bq m^-3^) in laboratory air at temperatures from 21.4 to 21.7°C and relative humidities from 26.6 to 29.0%. After the onset of stage 2, the placement of pitchblende and a beaker with 130 mL, 38°C warm water into the sealed terrarium strongly increased the abundance of negative ions in air (up to ~200,000 ions cm^-3^) and the concentration of ^222^Rn (~118 kBq m^-3^). The relative humidity exceeded 85%, and the air temperature intermittently rose by 5°C. The measurement uncertainty of the Canary Pro radon monitor increased with the ^222^Rn radiation level (Fig 4). However, the factory-documented uncertainty at the highest measured radiation level and the steadily increasing abundance of negative ions in air suggested that after a run time of ~17 h, the ^222^Rn-based radiation level exceeded 100 kBq m^-3^ (Fig 4; data shown in S1 File).

Experiment #2 at RHUL (Fig 2B) used the same sealed terrarium with pitchblende and high humidity to monitor and compare the CH_4_ mole fractions in the air of both the terrarium and the outside laboratory air over ~77 h. The Canary Pro radon monitor in the terrarium indicated an increase in ^222^Rn over time parallel to RHUL experiment #1. After 5 h into RHUL experiment #2, the ^222^Rn-based radiation in the terrarium was consistently > 50 kBq m^-3^. Despite high levels of ionization and ^222^Rn-based radiation in the terrarium, the CH_4_ mole fraction of 1.9941 ± 0.0036 ppm in terrarium air after being sealed for ~77 h was indistinguishable from the starting value of 1.9971 ± 0.0122 ppm within the uncertainty of measurements (Table 2).

**Table 2:** Results of experiment #2 at RHUL to assess the consumption of CH_4_ over time in the presence of elevated ^222^Rn concentrations and high relative humidity.

Date	Time	Sample	CH_4_ (ppm)		CH_4_ st. dev. (ppm)		H_2_0 (vol. %)	
March 4, 2016	11:00	Laboratory air	1.9760		0.0006		0.85	
March 4, 2016	11:15	Initial terrarium air	1.9971		0.0122		1.75	
March 4, 2016	13:15	Laboratory air	1.9616		0.0005		0.81	
March 4, 2016	13:15	Terrarium air	1.9873		0.0066		1.10	
March 4, 2016	16:00	Laboratory air	1.9643		0.0006		0.85	
March 4, 2016	16:00	Terrarium air	1.9818		0.0054		1.64	
March 7, 2016	16:05	Laboratory air	1.9693		0.0007		0.77	
March 7, 2016	16:05	Final terrarium air	1.9941		0.0036		1.84	

### Synopsis of combined results

The absence of any experimental evidence for accelerated loss of CH_4_ in the presence of elevated radiation makes it highly unlikely that radiation from radon isotopes is important in nature where ^220^Rn and ^222^Rn concentrations are typically much lower. Our data indicate that natural radiation in cave air cannot be responsible for the rapid consumption of CH_4_ in air on time-scales of days, even in caves with high relative humidity. The same conclusion had been reached from earlier laboratory experiments [18] and from observations in Australian cave air [15].

Many caves experience seasonally different degrees of venting and even reversals of air flow, which results in differences in air temperature and humidity and is difficult to simulate in laboratory experiments. Still, most cave environments at sufficient distances from cave entrances and vent holes are thermally buffered by surrounding rock and therefore do not express the relatively high diurnal and seasonal temperature and humidity variations as outside environments. Our experiments in laboratories were conducted at relatively constant room temperatures similar to many cave environments. Room temperatures in air-conditioned laboratory buildings are similar to actual temperatures in sub-tropical and tropical caves [14]. The use of water and moist soil in many of our experiments simulated the range of humidity in natural cave air. One possible caveat in terms of dissimilarity between our laboratory settings and actual caves may be the fact that our experiments allowed daylight to reach our experimental setups. However, the amount and timing of indirect light (no direct sunshine) was insufficient to let any photoautotrophs (algae) observably grow in our experiments. A necessary difference between air in our experiments at IU and actual cave air was the presence of traces of CH_4_ in our experiments. Some CH_4_ was needed to test for possible radiolytic destruction of CH_4_. In contrast, most natural cave air is depleted in CH_4_ relative to outside air. We conclude that the experimental conditions during experiments at IU and RHUL were reasonable approximations to simulate cave conditions. In the open atmosphere, solar radiation is mainly responsible for the generation of OH• radicals ([6], and refs. therein) that are the longest-lived potential radical reactant with CH_4_ in air. Subterranean radiolysis by radioactivity involves far more energy than photochemical dissociation of molecules by solar radiation, hence the speciation of resulting ions and radicals is different. A host of highly energetic, short-lived ions and radicals other than OH• is generated in subterranean air. The first abstraction of an H atom from CH_4_ requires a far higher activation energy than those of H atoms from methyl CH_3_ and methylene CH_2_ moieties. We argue that cave environments with elevated radioactivity may host short-lived, yet highly energetic radicals and ions that can supply the needed activation energy for first H-abstraction from CH_4_ more efficiently than OH• in the open atmosphere. Thus, the application of kinetic and energetic findings of photochemical CH_4_ oxidation in the open atmosphere may not be warranted for subterranean environments.

The α-radiation level in cave air is typically higher than in the open atmosphere because cave air is relatively close to rock and sediment surfaces with minerals harboring radioactive nuclides. The ionization rate in air *via* ^222^Rn radon decay is larger close to the ground, as reported for a Finnish forest [33], a spa [34], and in houses [35]. The effect is due to (i) strongly elevated radon concentrations in the air in porous, uranium-containing substrates and the rapid dilution of radon above surfaces upon mixing with the open atmosphere, especially during windy conditions. In contrast, cave air far from cave entrances is typically less turbulent and allows for a more even distribution of ^222^Rn in cave air. (ii) Short-lived ^220^Rn will always exhibit a greater abundance in air close to its parent nuclides in soil, rock, cave walls and floors [19]. Regardless, even exceptionally high combined radiation levels of ^220^Rn and ^222^Rn provided no evidence for accelerated CH_4_ oxidation in our experiments.

A plausible reason for slow radiolytic reaction kinetics is the mismatch between the large number of CH_4_ molecules in 1 m^3^ of atmosphere containing 1.85 ppmv CH_4_ at standard conditions (i.e., ~4.55 · 10^19^ molecules CH_4_) relative to the small number of radon-related nuclear decay events in the same volume of air (e.g., 10 kBq m^-3^ from ^222^Rn resulting from the decay of 10,000 atoms of ^222^Rn per second). The following simplistic numerical example illustrates the lack of feasibility of radiation-induced rapid oxidation of CH_4_. If we assume that 1 m^3^ of atmosphere entering a cave with 10 kBq m^-3^, even if every decay of ^222^Rn leads to the oxidation of one molecule CH_4_, it would require a geologic time period of ~144 million years to oxidize all CH_4_. In reality, the nuclide-specific radiation from the decay of ^222^Rn alone is dwarfed by the total radiation from radon, thoron, their radioactive progeny, and any other radioactive nuclides present in a given environment [19]. S1 File offers alternative calculations based on the assumptions that either (i) all energy from α-decay is exclusively invested in radiolytic dissociation of CH_4_ and results in the oxidation of multiple molecules of CH_4_ per decay event, or (ii) that only a fraction of the energy from α-decay is dissociating CH_4_ in the overwhelming presence of other molecules and atoms. The calculated time periods needed to degrade 1.85 ppmv CH_4_ at a ^222^Rn radiation level of 10 kBq m^-3^ range from 45.1 to 153,000 years, respectively. Even the most optimistic assumptions cannot speed up the radiolytic reaction kinetics to consume atmospheric CH_4_ within hours to days.

We can use the most optimistic scenario for consumption of 1.85 ppmv CH_4_ during 45.1 years at 10 kBq m^-3^ and calculate a radiation level of ~165 MBq m^-3^ that would be required to perform the same task in 24 h, which would be commensurate with kinetic CH_4_ observations in caves. Natural radiation levels of a few MBq m^-3^ have been measured in air where ^222^Rn emanates through geologic faults from underlying uranium minerals [36]. Radiation levels in the range of MBq m^-3^ have been observed in the air of uranium mines [27]. Still, no location is known to offer values close to the required ~165 MBq m^-3^. We conclude that there is no natural cave environment on earth where the α-radiation level is strong enough to rapidly degrade CH_4_. The same conclusion was recently described in a study that included arguments based on radiolytic kinetics of ion-induced reactions [15] that complement our calculations using α-decay and activation energy.

Subterranean radiation does not provide a mechanism for a fast-acting sink of atmospheric CH_4_ that would extend to arid and hyperarid environments, unlike microbial methanotrophy. Our study does not invalidate the geochemical data from previous studies documenting CH_4_ dynamics in subterranean ecosystems [10]. We do not call into question the fundamental importance of radiolysis of H_2_O (and other air components) and subsequent redox reactions that are documented in the geologic record (e.g., [37]) or the long-term subterranean radiolytic impact on sedimentary organic matter [1]. However, the exceedingly slow chemical rates of reaction caused by natural rates of radiolysis would likely take years to geologic time periods in cave environments to deplete trace amounts of atmospheric CH_4_ in cave air. As long as no alternative mechanisms have been identified, microbial methanotrophy serves as the only known fast-acting sink for subterranean CH_4_ in the critical and vadose zones.

## Conclusions

Strong radiation from radon isotopes and subsequent radiolysis of air proved unable to rapidly oxidize methane in dry or moist air. In the absence of a feasible alternative methane oxidation mechanism other than microbial methanotrophy, further studies are needed on the ability of microbes to consume trace amounts of methane in poorly ventilated caves, even though the trophic and energetic benefits become marginal at very low partial pressures of methane.

## Supporting information

S1 File An Excel file contains a first sheet “read me” with instructions and an overview on additional sheets offering analytical details and radiolysis calculations. (XLSX)
